# The utility of resilience as a conceptual framework for understanding and measuring LGBTQ health

**DOI:** 10.1186/s12939-016-0349-1

**Published:** 2016-04-06

**Authors:** Emily Colpitts, Jacqueline Gahagan

**Affiliations:** Gender and Health Promotion Studies Unit, School of Health and Human Performance, Dalhousie University, Stairs House, P.O. Box 15000, 6230 South Street, Halifax, N.S B3H 4R2 Canada

**Keywords:** LGBTQ, Health promotion, Health measurement, Resilience, Health research

## Abstract

**Background:**

Historically, lesbian, gay, bisexual, transgender and queer (LGBTQ) health research has focused heavily on the risks for poor health outcomes, obscuring the ways in which LGBTQ populations maintain and improve their health across the life course. In this paper we argue that informing culturally competent health policy and systems requires shifting the LGBTQ health research evidence base away from deficit-focused approaches toward strengths-based approaches to understanding and measuring LGBTQ health.

**Methods:**

We recently conducted a scoping review with the aim of exploring strengths-based approaches to LGBTQ health research. Our team found that the concept of resilience emerged as a key conceptual framework. This paper discusses a subset of our scoping review findings on the utility of resilience as a conceptual framework in understanding and measuring LGBTQ health.

**Results:**

The findings of our scoping review suggest that the ways in which resilience is defined and measured in relation to LGBTQ populations remains contested. Given that LGBTQ populations have unique lived experiences of adversity and discrimination, and may also have unique factors that contribute to their resilience, the utility of heteronormative and cis-normative models of resilience is questionable. Our findings suggest that there is a need to consider further exploration and development of LGBTQ-specific models and measures of resilience that take into account structural, social, and individual determinants of health and incorporate an intersectional lens.

**Conclusions:**

While we fully acknowledge that the resilience of LGBTQ populations is central to advancing LGBTQ health, there remains much work to be done before the concept of resilience can be truly useful in measuring LGBTQ health.

## Introduction

The concept of resilience is becoming increasingly prevalent in research focused on lesbian, gay, bisexual, transgender, and queer (LGBTQ) health. Though there is no widely agreed-upon definition of resilience, it commonly refers to the ability to withstand or overcome significant stress or adversity [1]. Evidence supports the notion that LGBTQ populations’ health outcomes are not necessarily a result of intrinsic individual-level characteristics indicating a lack of resilience; rather, health inequities and poor health outcomes among LBGTQ populations are a result of the adversity experienced by gender and sexually minoritized populations [2, 3]. For example, the ways in which LGBTQ health is often conceptualized and measured from a deficit-focused framework can have significant implications for health care access and uptake among LGBTQ populations, as the following section will discuss. LGBTQ health research has an important role to play in shifting the way that LGBTQ health is understood and measured in health policy and practice, which in turn has significant implications for health promotion strategies targeted at keeping LGBTQ populations healthy across the life course. The following section offers an overview of the key considerations in both understanding and measuring LGBTQ health.

## Background

The health needs and experiences of LGBTQ populations have generally been rendered invisible in mainstream health care systems and policies [4, 5]. This is, in part, because LGBTQ health has traditionally been understood through a heteronormative framework whereby the health needs and experiences of LGBTQ populations are assumed to be similar to those of their age-matched heterosexual and/or cisgender peers [4, 6, 7]. The invisibility of LGBTQ health needs and experiences has significant implications in terms of the provision of evidence-based, culturally competent health care. For example, heterosexist institutional systems, including health care systems, are comprised of heteronormative and cis-normative foundations, in that they presume that an individual is both heterosexual and cisgender, meaning that a persons’ gender identity is congruent with their sex assigned at birth (i.e. not transgender). These presumptions contribute to what Bauer et al. describe as the informational and institutional erasure of trans people in health care systems, which, in turn, results in systemic barriers to care [5]. For example, the Virginia Transgender Health Initiative Study found that the health care system was the most commonly cited area where transgender individuals experienced discrimination [8]. A study of LGBTQ patients’ experiences with medical intake forms found that the language and structure of intake forms tended to be overwhelmingly heteronormative and cis-normative, and thereby alienating to LGBTQ populations whose identities and lived experiences were not reflected in the forms [9]. These experiences of discrimination and informational and institutional erasure [5] can affect the ways in which LGBTQ populations choose to access or avoid health care services [4, 10, 11].

Public health policy and programming interventions have traditionally focused on individual-level indicators of health and on reducing the risk for negative health outcomes by changing individual, ‘lifestyle’ behaviour such as diet, exercise, and drug and alcohol use [4]. While not unique to public health policy and programming for LGBTQ populations, individual-level focus also contributes to the erasure and invisibility of LGBTQ health needs and experiences, which are heavily shaped by broader structural, systemic and social determinants of health [4]. For example, existing LGBTQ health research has demonstrated that social stigma, discrimination and victimization experienced by LGBTQ populations may affect uptake rates of preventative health screening programs and health care services [12–14]. It is equally important to note that LGBTQ populations may also experience negative determinants of health such as homelessness, social exclusion and poverty at higher rates than their age-matched heterosexual and/or cisgender peers [15–17]. The emphasis on individual-level determinants of health therefore obscures the broader structural and social determinants of LGBTQ health. Moreover, similar to non-LGBTQ populations, the overemphasis on individual-level determinants of health obscures the ways in which population-based supports work to reinforce the resilience demonstrated by LGBTQ populations and offer potential inroads for targeted health promotion strategies. For example, Herrick et al. argue that population-based initiatives that facilitate ‘coming out’ without fear of marginalization or violence are central to promoting the health of LGBTQ populations across the life course [3].

LGBTQ health research has a significant role to play in shifting how LGBTQ health is understood and measured, and, more specifically, the ways in which health research evidence is used to inform health policy and practice. However, given the longstanding focus on the risks for poor health outcomes among LGBTQ populations, including rates of sexually-transmitted infections (STI) and human immunodeficiency virus (HIV) infection, smoking, obesity and depression/suicidal ideation [18–20], a conceptual shift toward health-promoting LGBTQ research approaches is warranted. While deficit-focused health research is critical in the identification, mitigation and treatment of poor health outcomes among LGBTQ populations, it can also serve to reinforce negative perceptions of LGBTQ health. Moreover, deficit- and risk-focused research on LGBTQ health obscures the ways in which LGBTQ populations maintain their own health and avoid negative health outcomes. In response to the overemphasis on health research that takes as its starting point the risks and deficits among LGBTQ populations, health researchers have recently called for a shift in the focus of LGBTQ health research toward a more holistic understanding of LGBTQ health across the life course [21–23]. In this regard, it is important to explore LGBTQ health from a life course perspective, which considers the historical and social contexts that shape LGBTQ experiences [24]. For example, LGBTQ individuals who came of age when homosexuality was considered a psychiatric disorder or an illegal behaviour have very different experiences than younger LGBTQ individuals [24]. Moreover, there is an urgent need to move away from risk- and deficit-focused approaches toward strengths-based approaches to measuring and understanding LGBTQ health [25–27].

The emergence of health promotion approaches provides a promising conceptual shift in understanding and addressing LGBTQ health needs [28–30]. According to the World Health Organization, health promotion approaches focus on the “…process of enabling people to increase control over, and improve, their health”, which includes “a wide range of social and environmental interventions” [31]. It is important to note that health promotion recognizes the significance of both modifiable and non-modifiable determinants of health [32, 33] and emphasizes upstream, preventative approaches, which include the development of healthy public policy, in contrast to deficit-focused approaches [32]. Importantly, health promotion approaches have the potential to contribute to culturally and contextually meaningful health resources, which can contribute to resilience [34] among LGBTQ populations. Recognizing, rendering visible, and appropriately measuring the determinants of LGBTQ health and wellbeing is critical to the development of culturally competent health care services, systems, and policies for LGBTQ populations [21, 35].

### Current study

This paper draws on the findings of a recent scoping review, which sought to answer the following research question: what strengths-based approaches have been used to understand and measure LGBTQ health? The scoping review is part of a broader program of research on pathways to health among LGBTQ populations in Nova Scotia, Canada. Currently, there is no available data on the percentage of the population of Nova Scotia that identifies as LGBTQ and no baseline measurement of LGBTQ health in Nova Scotia. This is particularly concerning given that populations in the Atlantic region, including Nova Scotia, tend to have worse health outcomes than populations living in other parts of Canada [36]. As such, this program of research seeks to understand, measure and thereby render visible the health needs, outcomes and experiences of LGBTQ populations in Nova Scotia from a strengths-based perspective.

This paper is a broader exploration of resilience, which emerged as a key conceptual framework in our scoping review. Specifically, the purpose of this paper is to explore the utility of resilience as a conceptual framework in understanding and measuring LGBTQ health, with reference to the scoping review findings. Our findings have important implications for future strength-based research on LGBTQ health both in Nova Scotia and more broadly.

## Methods

Scoping reviews are a useful method of retrieving literature on a specific topic of interest or identifying gaps in the existing literature [37]. Our scoping review followed the methodology set out by Arksey and O'Malley, which involves six stages: identifying the research question; searching for relevant studies; study selection; charting the data; collating, summarizing, and reporting the results; and consulting with stakeholders. [37]. The focus of our scoping review was on identifying strengths-based approaches to LGBTQ health research. The key terms for the scoping review were determined by a community advisory committee comprised of LGBTQ community members, representatives from LGBTQ organizations, health researchers and a health reference librarian. In total, the scoping review, which was conducted in October 2014, yielded 1,855 de-duplicated results, of which 105 met the inclusion criteria (see Table 1 for the databases, key terms, and inclusion and exclusion criteria). Given the Canadian context of our research, we included only peer-reviewed strengths-based studies on LGBTQ health conducted in Canada, the United States, the United Kingdom, Australia and New Zealand in our review. A number of key conceptual and methodological frameworks were identified in the scoping review, including resilience, intersectionality, community-based participatory research, social ecology, and life course approaches. This paper will focus specifically on how the concept of resilience was defined and measured in studies included in the scoping review to examine its utility as a conceptual framework for strengths-based research on LGBTQ health.

**Table 1:** Scoping review search strategy

Criteria			
Search terms	Concept 1: LGBTQ identity	Concept 2: Health	Concept 3: Measurement
	Two spirit, LGB*, Lesbian*, Gay*, Transgender*, Transsexual*, homosexual, intersex*, gender minorit*, Queer*, Genderqueer, Gender varian*, Trans gender*, Trans sexual*, sexual minorit*	Resilienc*, Protective factor*, Health promot*, Health protect*, Life course*, Harm reduction, Health predict*, Social determinants of health, health disparities, Health status	Data collection, Survey*, Model*, Framework*, Measure*, Tool*, Assess*, Epidemiology, Module, Evaluat*
Databases	PubMED; CINAHL; PsychINFO; Gender Studies Database; History of Science, Technology and Medicine		
Inclusion	Published in English; Peer-reviewed; Academic journal; Primary study; study conducted in the US, UK, Australia, New Zealand or Canada; Strengths-based/health promotion perspective		
Exclusion	Published in language other than English; Non Peer-reviewed; Book, dissertation, conference abstract etc.; Not a primary study; Study conducted in country other than US, UK, Australia, New Zealand or Canada; deficit/risk-focused perspective		
Time Frame	The scoping review was conducted in October 2014. All included results were published before then. We did not limit our search using a start year.		

## Results and discussion

### Defining resilience

While there is no universally agreed-upon definition of resilience, one of the most common definitions cited in the articles included in the scoping review was from Luthar, Cicchetti and Becker, who define resilience as a “dynamic process encompassing positive adaptation within the context of significant adversity” (p. 543) [38]. Others characterized resilience as the ability to ‘bounce back’ from negative or challenging experiences [39, 40]. Singh and McKleroy define resilience as “a set of learned behaviours and interpersonal relationships that precedes one’s ability to cope with adversity” (p. 34) [41]. Herrick et al. argue that although some definitions describe resilience as an inherent personal trait, resilience should be understood as a process that develops and evolves across the life course [42].

Several definitions suggest that resilience is comprised of individual characteristics that serve as protective factors in coping with difficult situations [43, 44]. Others define resilience more broadly to include social and cultural capacities or resources in addition to individual-level factors [45–47]. Mutchler et al. argue that resilience must be understood as “referring both to individual characteristics and social mechanisms that support such characteristics” (p. 41) to avoid, for example, pathologizing sexual risk behaviour [48]. Smith and Gray contend that there are three factors of resilience development: supportive environments, protective interpersonal relationships and intrapersonal characteristics [27]. However, they caution that of these three factors, environments and interpersonal relationships are the areas where LGTBQ populations are most likely to experience stigma and discrimination, which they argue serves to justify their focus on individual-level factors of resilience.

### Multi-level factors contributing to measures of resilience

The articles included in the scoping review discuss and measure a wide range of factors potentially considered to contribute to resilience among LGBTQ populations. Factors at the individual level included positive self-esteem, self-efficacy, cognitive ability to mediate stress, self-acceptance, pro-active coping, self-care, shamelessness, and spirituality [43, 49–51]. Broader interpersonal, community and environmental factors included perceived social support, social connectedness, positive LGBTQ role models, positive representation of LGBTQ populations in the media, family acceptance, positive school and/or work environments, having access to safe spaces, connection to LGBTQ communities, and social activism [29, 41, 48, 49, 51–53]. Kubicek et al. conducted a study on the involvement of African American men who have sex with men in communities wherein individuals of diverse sexual and gender identities compete in events focused on dance, athletics and gender expression within a Ballroom subculture [26]. They found that Ballroom subculture serves as a protective environment and contributes to the resilience of young men who have sex with men. Reisner et al. argue that it is important to examine resilience resources, which they define as the contextual systems that affect individual-level health-promoting factors such as coping skills and self-efficacy [53]. They assert that these health-promoting factors are likely to be most effective in the context of a supportive environment.

### Models for operationalizing resilience as a measurement tool

The majority of the models used in the studies stem from the broader resilience literature and are not specific to LGBTQ populations. For example, in their study on the resilience of black lesbians, Bowleg et al. employ Kumpfer’s transactional model, which involves six predictors of resilience: stressors, external environmental contextual factors, person-environment interactional processes, internal psychological factors, stress coping processes developed through exposure to stress, and positive life outcomes [54, 55]. Gamarel et al. draw on a relational health model developed at the Stone Center at Wellesley College in Massachusetts as the basis for their conceptualization of resilience [56]. This model involves four aspects of relationships that enhance resilience: empowerment, authenticity, perceived mutual engagement, and conflict tolerance. Mustanski et al. employ a variable-centred resilience model, which examines the variability of a negative outcome based on health-promoting factors, to measure the relationship between psychological distress among LGBTQ youth and peer and family support [57].

Herrick et al. argue that resilience can be conceptualized through three different models [42]. The first is a compensatory model, wherein protective factors are associated with positive outcomes. The second is a protective model, wherein protective factors buffer the relationship between risk and the outcome. The third is a challenge model, which describes a curvilinear relationship between risk and negative health outcomes. At high and low levels of risk, there is a positive relationship to negative health outcomes whereas individuals with moderate levels of risk “have been exposed to just enough risk so that they have learned how to cope with or avoid the associated outcome, but not so much so that they can no longer cope” (Herrick et al. 2014, p. 5) [42]. Herrick et al. also argue that the challenge model may be particularly well-suited for LGBTQ populations because they face constant, institutionalized adversity such as homophobic or transphobic discrimination [42]. In this regard, Herrick et al. suggest that aspects of LGBTQ resilience may conform to any or all of these models at a given moment depending on the timing, the individual developmental stage and a variety of other factors [42].

Other health researchers have argued that resilience among LGBTQ populations must be understood and measured through LGBTQ-specific resilience models rather than extrapolating from existing models and measures. For example, Smith and Gray explain that while LGBTQ populations may be very resilient, they generally perform poorly on existing psychometric measures of resilience [27]. Based on this observation, they argue that these instruments fail to adequately measure the strengths and resilience of LGBTQ populations, thereby perpetuating the emphasis on risks and deficits. As such, Smith and Gray developed an LGBTQ-specific rapid assessment instrument to measure personal hardiness, which they define as a critical aspect of resilience [27]. Fenaughty and Harré also developed their own LGBTQ-specific model, the Seesaw Model, to capture the balance between risk and resilience factors related to LGB youth suicide [52]. They use the image of a seesaw to argue that the balance between risk and resilience is constantly in flux depending on the ‘weight’ of the risk and resilience factors present.

Other health research studies included in our scoping review, while not measuring resilience directly, measured factors potentially associated with resilience. Specifically, several employed an amalgam of standardized measures to assess potential factors of resilience such as self-esteem, perceived social support and proactive coping [49, 51, 57, 58]. The majority of standardized instruments used to understand and measure these factors are not LGBTQ-specific, although a few were adapted or modified for LGBTQ populations. In fact, the only instrument that measured resilience directly was Wagnild and Young’s 14-item Resilience Scale [59] used by King and Orel in their study of resilience among midlife and older gay men living with HIV/AIDS [44]. The Resilience Scale, while not LGBTQ-specific, measures the following five components: self-reliance, meaningfulness, perseverance, equanimity and existential aloneness [59].

### Conceptual and theoretical explorations

It is important to note that several other health research studies used qualitative methods to investigate resilience. For example, grounded theory, which allows “… for the ‘discovery’ of meaning and the generation of theory through systematic analysis of qualitative data that begins with minimal a priori assumptions” (Van Wagenen et al. 2013, p. 8) [30], was used in several of the resilience-focused studies included in the scoping review [26, 52, 60]. Similarly, Singh et al. used phenomenological approaches to explore the resilience of transgender individuals by asking questions such as “when you hear the word 'resilience', what words or phrases come to mind about your life experience as a transgender person?" and "over your life as a transgender person, when are times you felt more or less resilient?" (p. 22) [61]. Studies using qualitative methods such as these were generally focused on examining the relevance of resilience to the experiences of LGBTQ populations and how they defined and understood it from their own lived experience, rather than on quantitative measures of resilience.

The majority of resilience-focused articles included in our scoping review concluded that resilience may in fact be an appropriate and useful concept for understanding and measuring LGBTQ health. For example, in their study on successful aging, Fredriksen-Goldsen et al. argue that resilience is critical to understanding how older LGBTQ populations maintain quality of life and successful aging [47]. Further, other researchers suggest that understanding and measuring the resilience of LGBTQ populations is important because it represents a significant departure from the risk and deficit focus that LGBTQ health research has historically employed [62]. In fact, resilience-based health-related interventions were seen as being likely to have important impacts on LGBTQ health outcomes [27, 48, 62]. Herrick et al. argue that while risk-focused research can help identify important health issues among LGBTQ populations, it does not necessarily identify solutions aimed at addressing them at the systemic or population-based level [42]. Moreover, interventions that focus on enhancing the resilience of LGBTQ populations are more likely to have long-term sustainable impacts on LGBTQ health outcomes and related supports than interventions that focus merely on reducing individual risks [42]. The factors encompassed in the concept of resilience, broadly speaking, may be protective against a myriad of health risks faced by LGBTQ populations, while risk-based interventions often tend to focus on addressing risk for specific, individual-level health issues.

### Primary conceptual concerns with the operationalization of “resilience”

However, it is noteworthy that some LGBTQ health researchers continue to question the utility of resilience as a conceptual framework for a variety of reasons. In their study on LGBTQ mental health, Dickinson and Adams contend that resilience places an overemphasis on the individual level and is therefore limited as an approach to measuring and understanding LGBTQ health from a strengths-based or structural perspective [43]. Further, they argue that models which take as their starting point a more holistic approach and which place greater emphasis on broader social and structural factors are considered more appropriate for understanding and measuring LGBTQ health. Specifically, they suggest that a broader health promotion approach presents a useful alternative to resilience in isolation as it seeks to improve health and well-being at the individual, community and societal levels.

Another key concern regarding resilience as a conceptual framework in understanding and measuring LGBTQ health is that it has traditionally been framed and conceptualized from an ethnocentric, white, Western perspective, as the emphasis on individualism demonstrates [39, 41]. This culturally specific conceptualization of resilience may limit its effectiveness as a framework for understanding and measuring the health of diverse LGBTQ populations. In their study on resilience, stress and coping among selected non-dominant groups (including gay and lesbian populations), Iwasaki et al. found that the participants described their own resilience as being deeply connected to their cultures and/or sub-cultures [60]. Further, Iwasaki et al. argue that given the narrow and ethnocentric way in which resilience is currently typically understood, there is a need to develop more culturally appropriate resilience frameworks for LGBTQ populations [60]. Singh and McKleroy echo this suggestion based on the findings of their research on resilience among transgender persons of colour [41]. Specifically, these arguments relate to intersectionality, a theoretical approach that examines multiple and intersecting systems of privilege and oppression [63, 64]. Adopting an intersectionality lens allows for a more comprehensive understanding of how the health outcomes and resilience of LGBTQ populations are influenced by the intersections of LGBTQ identity and race and class, for example. It also emphasizes the fact LGBTQ populations must be understood as heterogeneous groups with diverse health needs, experiences, and outcomes [4]. As such, intersectionality must be taken into account when conceptualizing resilience [54].

## Conclusions

### Implications for future research

The purpose of this paper was to explore the utility of resilience as a conceptual framework in understanding and measuring the health of LGBTQ populations based on a scoping review of LGBTQ health literature from a strengths-based, health promotion perspective. The fact that there is no clearly agreed-upon definition of resilience presents a challenge in determining its utility for strengths-based LGBTQ health research. Further, the tendency for resilience to focus on individual-level factors or to be characterized as a set of inherent intrapersonal traits is particularly concerning in light of the ways in which privileging the individual over the structural and the social has contributed to the invisibility and erasure of LGBTQ health needs and experiences within health policy and health care systems. Broader definitions of resilience (see, for example, Ungar et al. [34]), which take into account structural, social, and individual determinants of health are more consistent with the ecological health promotion model. There is also a notable absence of baseline and longitudinal data on resilience, and future research on the utility of resilience in measuring LGBTQ health should consider how resilience develops and changes across the life course.

Given the diversity of factors potentially contributing to resilience as cited in the articles included in our scoping review, it is difficult to determine which factors are most relevant to promoting the health of LGBTQ populations in particular contexts, such as Nova Scotia. As such, in order to utilize resilience in understanding and measuring LGBTQ health, our health research approaches must first determine the key factors that contribute to resilience among LGBTQ populations. The debate on whether resilience models should be LGBTQ-specific is also critical in moving forward. LGBTQ populations have unique lived experiences of adversity and discrimination based on their interactions within heteronormative and cis-normative health and social care systems, which influence their pathways to health across the life course. In addition, LGBTQ populations may also have unique resilience factors that can promote and enhance health across the life course which need to be better understood and measured [65]. As such, the ongoing focus and utility of individual-level, mainstream, heteronormative and cis-normative models of resilience in understanding and measuring LGBTQ health is questionable. Finally, models of resilience must reflect and incorporate intersectionality. Incorporating an intersectional lens acknowledges the complex intersecting and compounding nature of marginalization, oppression, risk factors and their subsequent impacts of LGBTQ health across the life course.

In comparison with interventions focused on mitigating health risks, the potential impact of LGBTQ health interventions focused on promoting resilience in relation to health outcomes is promising. As such, there is a need for more comprehensive theoretical and conceptual models that include resilience in the future design of health promotion strategies. However, while we fully acknowledge the resilience of LGBTQ populations, we argue that there is much work to be done before it can be truly useful as a concept in measuring LGBTQ health.

### Limitations

The findings of our scoping review presented here and the subsequent conceptual mapping of resilience are subject to potential limitations. The databases used for this study were searched for English language, peer-reviewed, published articles only. Therefore, relevant data presented in a language other than English or that is not available in peer-reviewed academic literature may have been excluded. Given that our research is based in the context of Nova Scotia, Canada, only studies conducted in Canada, the United States, the United Kingdom, Australia and New Zealand were included in our scoping review. As such, there may be studies on resilience based in other contexts that were excluded. Further, given that this paper draws specifically on the articles included in the scoping review, there may be other research on resilience among LGBTQ populations that is not represented.

### Significance

This paper builds on existing knowledge on LGBTQ health by providing a review of studies that explore resilience among LGBTQ populations. Given the traditional emphasis on health risks and deficits among LGBTQ populations, our emphasis on strengths-based approaches to LGBTQ health, including resilience, is significant. Moreover, this paper builds on the broader literature on resilience by focusing on LGBTQ populations, which highlights the need to consider how resilience might be understood and measured differently for LGBTQ populations.

It is essential that we continue theoretical and conceptual exploration of resilience among LGBTQ populations. A more comprehensive understanding of LGBTQ resilience will allow us to gain a more comprehensive and holistic understanding of pathways to health among LGBTQ populations. It will also provide insight on relevant health interventions and health promotion strategies aimed at advancing LGBTQ health across the life course. Exploring approaches to LGBTQ health that are designed to not only address vulnerabilities but also to incorporate and support resilience has the potential to have a significant impact on the health outcomes of LGBTQ populations.

## Abbreviations

HIV: human immunodeficiency virus
LGBTQ: lesbian, gay, bisexual, transgender and queer
STI: sexually transmitted infection

## References

[1] Fergus S, Zimmerman MA (2005). Adolescent resilience: a framework for understanding healthy development in the face of risk. Annu Rev Public Health.

[2] Herrick AL, Lim SH, Plankey MW, Chmiel JS, Guadamuz TE, Kao U (2013). Adversity and syndemic production among men participating in the multicenter AIDS cohort study: a life-course approach. Am J Public Health.

[3] Herrick AL, Egan JE, Coulter RWS, Friedman MR, Stall R (2014). Raising sexual ninority youths' health levels by incorporating resiliencies into health promotion efforts. Am J Public Health.

[4] Mulé N, Ross L, Deeprose B, Jackson B, Daley A, Travers A, Moore D (2009). Promoting LGBT health and wellbeing through inclusive policy development. Int J Equity Health.

[5] Bauer GR, Hammond R, Travers R, Kaay M, Hohenadel KM, Boyce M (2009). “I don't think this is theoretical; this is our lives”: how erasure impacts health care for transgender people. J Assoc Nurses AIDS.

[6] Röndahl G (2011). Heteronormativity in health care education programs. Nurse Educ Today.

[7] Mayer K, Bradford J, Makadon H, Stall R, Goldhammer H, Landers S (2008). Sexual and gender minority health: what we know and what needs to be done. Am J Public Health.

[8] Bradford J, Reisner SL, Honnold JA, Xavier J (2013). Experiences of transgender-related discrimination and implications for health: results from the Virginia transgender health initiative study. Am J Public Health.

[9] Goins ES, Pye D (2013). Check the box that best describes you: reflexively managing theory and praxis in LGBTQ health communication research. J Health Commun.

[10] Ryan B, Chervin M (2000). Framing gay men's health in a population health discourse.

[11] Dean L, Meyer IH, Robinson K, Sell RL, Sember R, Silenzio VMB (2000). Lesbian, gay, bisexual, and transgender health: findings and concerns. J Gay Lesbian Med Assoc.

[12] Johnson CW, Singh AA, Gonzalez M (2014). "It's complicated": collective memories of transgender, queer, and questioning youth in high school. J Homosex.

[13] Makadon H (2011). Ending LGBT invisibility in health care: the first step in ensuring equitable care. Cleve Clin J Med.

[14] National Institutes of Health (2011). The health of lesbian, gay, bisexual, and transgender people: building a foundation for better understanding.

[15] Kitts R (2010). Barriers to optimal care between physicians and lesbian, gay, bisexual, transgender, and questioning adolescent patients. J Homosex.

[16] McBride DL (2012). Homelessness and healthcare disparities among lesbian, gay, bisexual and transgender youth. Pediatr Nurs.

[17] McKay B (2011). Lesbian, gay, bisexual, and transgender health issues, disparities, and information resources. Med Ref Serv Q.

[18] McGibbon E, Etowa J, McPherson C (2008). Health-care access as a social determinant of health. Can Nurse.

[19] Mereish E, O’Cleirigh C, Bradford JB (2014). Interrelationships between LGBT-based victimization, suicide, and substance use problems in a diverse sample of sexual and gender minorities. Psychol Health Med.

[20] Newcomb M, Heinz A, Birkett M, Mustanski B (2014). A longitudinal examination of risk and protective factors for cigarette smoking among lesbian, gay, bisexual, and transgender youth. J Adolesc Health.

[21] Berberet HM (2006). Putting the pieces together for queer youth: A model of integrated assessment of need and program planning. Child Welfare.

[22] Gardner AT, de Vries B, Mockus DS (2014). Aging out in the desert: disclosure, acceptance, and service use among midlife and older lesbians and gay men. J Homosex.

[23] Jenkins Morales M, King MD, Hiler H, Coopwood MS, Wayland S (2014). The greater St. Louis LGBT health and human services needs assessment: an examination of the silent and baby boom generations. J Homosex.

[24] Fredriksen-Goldsen KI, Simoni JM, Kim HJ, Lehavot K, Walters KL, Yang J (2014). The health equity promotion model: reconceptualization of lesbian, gay, bisexual, and transgender (LGBT) health disparities. Am J Orthopsychiatry.

[25] Herrick AL, Lim SH, Wei C, Smith H, Guadamuz T, Friedman MS, Stall R (2011). Resilience as an untapped resource in behavioral intervention design for gay men. AIDS Behav.

[26] Kubicek K, McNeeley M, Holloway IW, Weiss G, Kipke MD (2013). 'It’s like our own little world': resilience as a factor in participating in the ballroom community subculture. AIDS Behav.

[27] Smith MS, Gray SW (2009). The courage to challenge: a new measure of hardiness in LGBT adults. J Gay Lesbian Soc Serv.

[28] Carver C (1998). Resilience and thriving: issues, models, and linkages. J Soc Issues.

[29] Scourfield J, Roen K, McDermott L (2008). (2008) Lesbian, gay, bisexual and transgender young people's experiences of distress: resilience, ambivalence and self-destructive behaviour. Health Soc Care Community.

[30] Van Wagenen A, Driskell J, Bradford J (2013). ''I'm still raring to go'': successful aging among lesbian, gay, bisexual, and transgender older adults. J Aging Stud.

[31] World Health Organization. Health Promotion. http://www.who.int/topics/health_promotion/en/. Accessed on 7 July 2015

[32] Green J, Tones K, Cross R, Woodall J (2015). Health promotion: planning and strategies.

[33] Public Health Agency of Canada. What determines health? http://www.phac-aspc.gc.ca/ph-sp/determinants/index-eng.php. Accessed 7 July 2015.

[34] Ungar M, Brown M, Liebenberg L (2008). Cheung M & Levine K (2008). Distinguishing differences in pathways to resilience among Canadian youth. Can J Commun Ment Health.

[35] Brotman S, Ryan B, Cormier R (2003). The health and social service needs of gay and lesbian elders and their families in Canada. Gerontologist.

[36] Statistics Canada (2013). Health in Canada: health profile.

[37] Arksey H, O'Malley L (2005). Scoping studies: towards a methodological framework. Int J of Soc Res Meth.

[38] Luthar SS, Cicchetti D, Becker B (2000). The construct of resilience: a critical evaluation and guidelines for future work. Child Dev.

[39] Singh AA (2013). Transgender youth of color and resilience: negotiating oppression and finding support. Sex Roles.

[40] Rowan NL, Butler SS (2014). Resilience in attaining and sustaining sobriety among older lesbians with alcoholism. J Gerontol Soc Work.

[41] Singh AA, McKleroy VS (2011). "Just getting out of bed is a revolutionary act": the resilience of transgender people of color who have survived traumatic life events. Eur Phys Educ Rev.

[42] Herrick AL, Stall R, Goldhammer H, Egan JE, Mayer KH (2014). Resilience as a research framework and as a cornerstone of prevention research for gay and bisexual men: theory and evidence. AIDS Behav.

[43] Dickinson P, Adams J (2014). Resiliency and mental health and well-being among lesbian, gay and bisexual people. Int J of Ment Health Promot.

[44] King SD, Orel N (2012). Midlife and older gay men living with HIV/AIDS: the influence of resiliency and psychosocial stress factors on health needs. J Gay Lesbian Soc Serv.

[45] Follins LD, Walker JJ, Lewis MK (2014). Resilience in black lesbian, gay, bisexual and transgender individuals: a critical review of the literature. J Gay Lesbian Ment Health.

[46] Fredriksen-Goldsen K, Emlet CA, Kim H, Muraco A, Erosheva EA, Goldsen J (2013). The physical and mental health of lesbian, gay male, and bisexual (LGB) older adults: the role of key health indicators and risk and protective factors. Gerontologist.

[47] Fredriksen-Goldsen KI, Kim HJ, Shiu C, Goldsen J, Emlet CA (2015). Successful aging among LGBT older adults: physical and mental health-related quality of life by age group. Gerontologist.

[48] Mutchler MG, Ayala G, Neith KL (2005). Safer sex stories told by gay men: building resilience through gay-boy talk. J Gay Lesbian Issues Educ.

[49] Anderson AL (1998). Strengths of gay male youth: an untold story. Child Adolesc Social Work J.

[50] Bockting WO, Miner MH, Swinburne Romine RE, Hamilton A, Coleman E (2013). Stigma, mental health, and resilience in an online sample of the US transgender population. Am J Public Health.

[51] Craig SL, Austin A, McInroy LB (2014). School-based groups to support multiethnic sexual minority youth resiliency: preliminary effectiveness. Child Adolesc Social Work J.

[52] Fenaughty J, Harré N (2003). Life on the seesaw: a qualitative study of suicide resiliency factors for young gay men. J Homosex.

[53] Reisner SL, Biello K, Perry NS, Gamarel KE, Mimiaga MJ (2014). A compensatory model of risk and resilience applied to adolescent sexual orientation disparities in nonsuicidal self-injury and suicide attempts. Am J Orthopsychiatry.

[54] Bowleg L, Craig ML, Burkholder G (2004). Rising and surviving: a conceptual model of active coping among black lesbians. Cultur Divers Ethnic Minor Psychol.

[55] Kumpfer KL, Glantz MD, Johnson JL (1999). Factors and processes contributing to resilience: the resilience framework. Resilience and development: positive life adaptations.

[56] Gamarel KE, Walker JJ, Rivera L, Golub SA (2014). Identity safety and relational health in youth spaces: a needs assessment with LGBTQ youth of color. J LGBT Youth.

[57] Mustanski B, Newcomb ME, Garofalo R (2011). Mental health of lesbian, gay, and bisexual youths: a developmental resiliency perspective. J Gay Lesbian Soc Serv.

[58] Grossman AH, D'Augelli AR, Frank JA (2011). Aspects of psychological resilience among transgender youth. J LGBT Youth.

[59] Wagnild G, Young HM (1993). Development and psychometric evaluation of the resilience scale. J Nurs Meas.

[60] Iwasaki Y, Bartlett J, MacKay K, Mactavish J, Ristock J (2005). Social exclusion and resilience as frameworks of stress and coping among selected non-dominant groups. Int J Ment Health Promot.

[61] Singh AA, Hays DG, Watson LS (2011). Strength in the face of adversity: resilience strategies of transgender individuals. J Couns Dev.

[62] Herrick AL, Stall R, Chmiel JS, Guadamuz TE, Penniman T, Shoptaw S (2013). It gets better: resolution of internalized homophobia over time and associations with positive health outcomes among MSM. AIDS Behav.

[63] Hankivsky O, Christoffersen A (2008). Intersectionality and the determinants of health: a Canadian perspective. Crit Public Health.

[64] Crenshaw K (1991). Mapping the margins: intersectionality, identity politics, and violence against women of color. S. L. Rev..

[65] Russell ST (2005). Beyond risk: resilience in the lives of sexual minority youth. J Gay Lesbian Issues Educ.

